# NafA Negatively Controls *Neisseria meningitidis* Piliation

**DOI:** 10.1371/journal.pone.0021749

**Published:** 2011-07-01

**Authors:** Asaomi Kuwae, Hong Sjölinder, Jens Eriksson, Sara Eriksson, Yao Chen, Ann-Beth Jonsson

**Affiliations:** Department of Genetics, Microbiology and Toxicology, Stockholm University, Stockholm, Sweden; Health Protection Agency, United Kingdom

## Abstract

Bacterial auto-aggregation is a critical step during adhesion of *N. meningitidis* to host cells. The precise mechanisms and functions of bacterial auto-aggregation still remain to be fully elucidated. In this work, we characterize the role of a meningococcal hypothetical protein, NMB0995/NMC0982, and show that this protein, here denoted NafA, acts as an anti-aggregation factor. NafA was confirmed to be surface exposed and was found to be induced at a late stage of bacterial adherence to epithelial cells. A NafA deficient mutant was hyperpiliated and formed bundles of pili. Further, the mutant displayed increased adherence to epithelial cells when compared to the wild-type strain. In the absence of host cells, the NafA deficient mutant was more aggregative than the wild-type strain. The *in vivo* role of NafA in sepsis was studied in a murine model of meningococcal disease. Challenge with the NafA deficient mutant resulted in lower bacteremia levels and mortality when compared to the wild-type strain. The present study reveals that meningococcal NafA is an anti-aggregation factor with strong impact on the disease outcome. These data also suggest that appropriate bacterial auto-aggregation is controlled by both aggregation and anti-aggregation factors during *Neisseria* infection *in vivo.*

## Introduction

*N. meningitidis* is an obligate human pathogen and a causative agent of meningitis and sepsis. The reservoir for this microbe is the nasopharynx and up to 40% of some populations are asymptomatic carriers [1]. Occasionally bacteria can gain access to the bloodstream and spread to the cerebrospinal fluid, causing sepsis and meningitis. The prerequisite for meningococcal disease is bacterial colonization of the nasopharyngeal mucosa. *N. meningitidis* uses a two-step strategy to adhere to host cells, with an initial attachment phase followed by an intimate adherence phase.

Initial attachment of bacteria to cells is mediated by the type IV pili (TFP) that extend from the bacterial surface [2], [3], [4], [5]. TFP are thin hair-like filaments and are responsible for several virulence-associated functions such as twitching motility, microcolony formation, and auto-aggregation, which in turn are essential properties for efficient initial bacteria-cell interaction (reviewed in [6]). TFP are distributed widely among commensal, environmental and pathogenic bacteria, such as enteropathogenic *E. coli*, *Pseudomonas aeruginosa*, *Myxococcus xanthus* and *Haemophilus influenzae*. The importance of auto-aggregation in *Neisseria* infection has been previously investigated by inactivation of one of the minor pilins, PilX, which is necessary for pilus-pilus interaction. PilX-deficient strains are unable to form microcolonies and show defects in adherence to cultured mammalian cells [7]. The aggregative properties of PilX have been linked to a “hook” domain in the C-terminus that is essential for pilus-pilus (PilX-PilX) interaction [8]. After initial attachment, pili are down-regulated and microcolonies begin to disperse. The bacteria then spread onto the cell surface as a monolayer, which initiates intimate adherence [9]. It has been suggested that down-regulation of pili and microcolony dispersal is mediated by the ATPase PilT [10].

Several bacterial membrane-associated components are involved in intimate bacteria-cell interaction, such as lipooligosaccharides (LOS), the capsular polysaccharides, and outer membrane proteins including opacity proteins (Opa) and the Opc protein. LOS is an important inducer of the host inflammatory response in meningococcal sepsis [11], [12]. The capsular polysaccharides are needed for septicaemia and meningitis [13] and are important factors for intracellular meningococcal survival [14]. It has been shown that down regulation of capsule is associated with intimate adhesion of meningococci to host cells [15], [16]. *N. meningitidis* typically expresses four kinds of opacity proteins that interact with host cells during intimate adherence. The Opa proteins directly interact with the CEACAM (carcinoembryonic antigen-related cell adhesion molecule) family of receptors [17], [18], [19]. The Opc protein forms a complex with host integrins through fibronectin or vitronectin [20], [21]. Furthermore, Opa and Opc proteins have been linked with binding to heparan sulphate proteoglycans [18], [22], [23].

In this study, we investigated the role in pathogenesis of a conserved bacterial protein, here named NafA, which is up-regulated and surface expressed following adhesion of *N. meningitidis* to host cells. NafA (NMB0995) was previously reported as a potential vaccine candidate component for serogroup B strains [24]. We demonstrate that NafA acts as an anti-aggregation factor at the bacterial surface preventing excess auto-aggregation and microcolony formation during colonization of epithelial cells. Furthermore, the anti-aggregation property mediated by NafA is shown to enhance *N. meningitidis* virulence in a murine disease model.

## Results

### NafA is 19 kDa and highly conserved among *Neisseria* species

**Figure 1:**
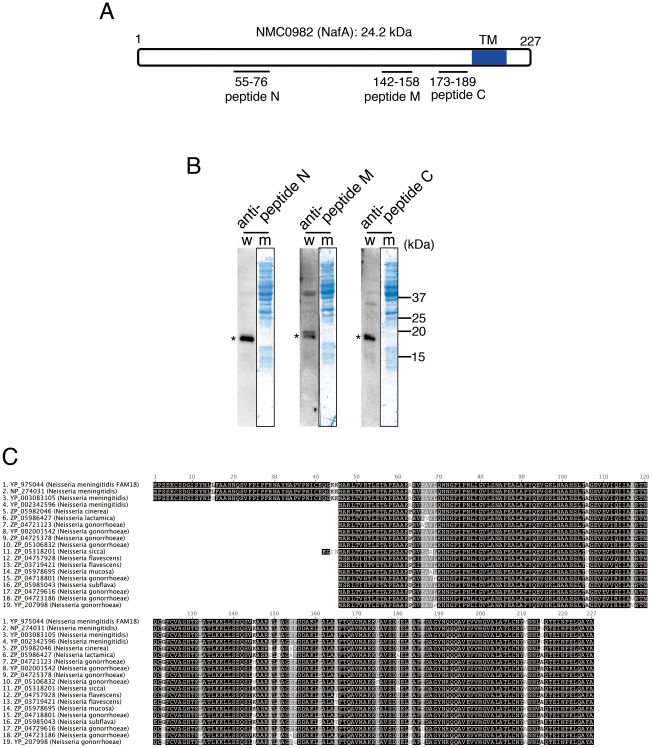
NafA is a highly conserved protein among *Neisseria* species. A. Schematic representation of NMC0982 (NafA). Regions corresponding to the three peptides used as immunogens are indicated. A transmembrane domain (TM) predicted by the TMpred program (http://www.ch.embnet.org/software/TMPRED_form.html) is indicated as blue colour at the C-terminal. B. NafA was detected in whole cell lysate of FAM20 wild-type strain by western blot (w) using each peptide-generated antibody. The specific signal of NafA is indicated with an asterisk. Developed membranes (m) stained with amino black are also shown. C. BLAST alignment of NafA homologues from 19 different *Neisseria* strains and species, ordered by identity. The identity between NMC0982 (1. YP_975044) and NGO0887 (19. YP_207998) is 90.1%.

In recent years, genomic and transcriptional studies of pathogenic bacteria have highlighted a large set of hypothetical proteins, to which specific biological functions have not been allocated. One such protein is hypothetical protein NMB0995 (NCBI Reference Sequence: NP_274031.1) in *N. meningitidis* serogroup B strain MC58. NMB0995 is here named NafA for *Neisseria* anti-aggregation factor A (see below). It has been shown that NafA displays low levels of genetic variation in tested meningococcal strains, is up-regulated in FACS analyses upon bacterial adhesion to host cells, and that murine antibodies raised against full-length NafA are bactericidal against *N. meningitidis* [24]. To investigate the role of NafA in meningococcal virulence, we generated and purified rabbit antibodies against three regions of NafA (Fig. 1A). Whole cell lysates of *N. meningitidis* serogroup C strain FAM20 were analysed by western blotting using the purified anti-NafA antibodies. The western blot results indicated a specific protein band with a molecular mass of 19 kDa (Fig. 1B), whilst the expected theoretical size of NafA was 227 amino acids, *i.e.* 24.2 kDa (Fig. 1A). Therefore interspecies NafA reference sequences were analyzed and aligned (Fig. 1C). The protein was found to be highly conserved among different *Neisseria* species, including the pathogen *N. gonorrhoeae*, and the human specific commensal species such as *N. sicca*, *N.cinerea*, *N. lactamica*, *N. flavescens*, *N. mucosa*, and *N. subflava* (Fig. 1C). The sequence identity among reported NafA homologues is at least 90%. NafA of MC58, *i.e*. NMB0995 (2. NP_274031 in Fig. 1C) and NafA of serogroup C FAM18 strain, *i.e.* NMC0982 (1. YP_975044 in Fig. 1C) share 99.6% sequence identity with a predicted 227 amino acid coding region corresponding to a theoretical molecular mass of 24.2 kDa. The sequence corresponding to the first 45 N-terminal residues was absent from 3 of 19 neisserial sequences but present in 3 of 4 meningococcal sequences, suggesting a miss-annotation of these sequences. Taken together, these results strongly suggest that native NafA is 182 amino acids long with the actual start codon at amino acid 46 of the originally annotated sequence, and not 227 amino acids as previously reported.

### NafA is surface expressed upon meningococcal attachment to human cells

**Figure 2:**
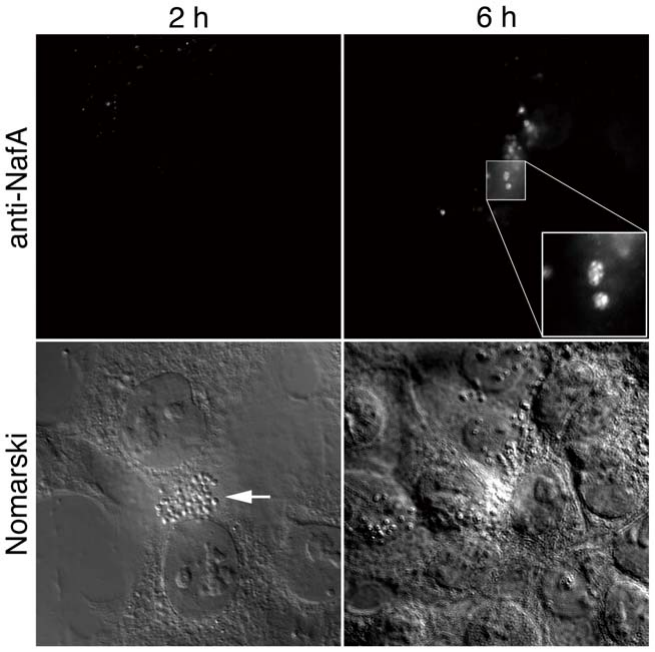
NafA is induced and exposed at the bacterial surface upon host cells interaction. FaDu cells were infected with the wild-type strain for 2 h (left column) or 6 h (right column). After washing away unbound bacteria, the cells were stained with anti-NafA peptide N antibody. Fluorescent (upper row) and Nomarski (lower row) images are shown. A 100 x objective lens was used. The inset shows higher magnification of the boxed area in the image. Arrow indicates a microcolony on the cell.

Several lines of evidence have demonstrated that expression of certain meningococcal outer membrane components is regulated upon contact with the host epithelium, thereby facilitating meningococcal colonization [15], [25]. NafA was previously detected on the bacterial surface by flow cytometry after ethanol permeabilization of the bacterial capsule [24]. To further monitor regulation of NafA and to visually determine its localization during bacteria-host cell interaction, we infected the human pharyngeal epithelial cell line FaDu with *N. meningitidis* FAM20 for 2 h and 6 h and analyzed the NafA expression by fluorescence microscopy using an anti-NafA antibody. At an early adhesion stage, *i.e.* 2 h post infection, we could not observe any specific staining (Fig. 2). However, at 6 h post infection, specific fluorescent signal of NafA could clearly be detected at the bacterial surface (Fig. 2, Fig. S4). These results argue that NafA becomes surface exposed at a later stage of bacterial adhesion to host cells, *i.e.* after 6 h of infection.

### NafA affects bacterial piliation

**Figure 3:**
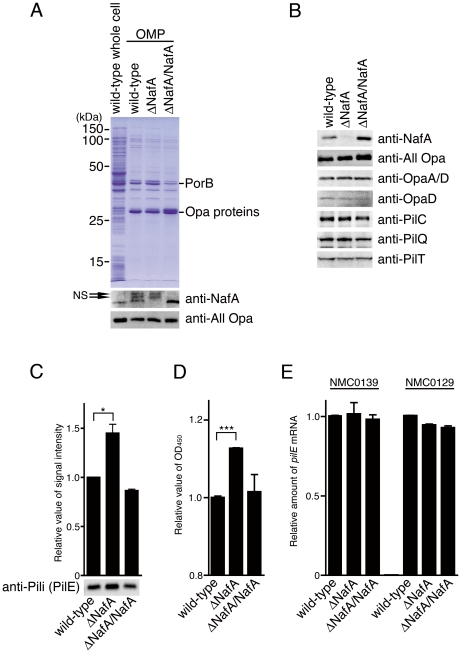
The ΔNafA strain expresses more PilE protein than the wild-type strain. Bacterial outer membrane proteins (A) and whole cell lysate samples (B) were prepared from strains indicated and separated by SDS-PAGE. A. Expression profiles of outer membrane protein were detected by Coomassie Brilliant Blue staining. PorB and Opa proteins were confirmed by MALDI-TOFMS. The lower panel shows the expression of NafA and Opa proteins in the outer membrane fraction, as detected by western blot analysis using anti-NafA peptide C antibody and anti-All Opa antibody, respectively. Arrows indicate non-specific bands (NS). B. Detection of NafA, Opa proteins and pilus components from whole bacterial lysate samples by western blot analysis. Equal amounts of whole cell lysates were loaded in each western blot analysis. C. The western blot analysis was performed as panel B. The signal intensities were analyzed using ImageJ software and relative value were presented when value of the wild-type strain was set as 1.0. D. Amounts of PilE produced by indicated strains were compared by whole cell ELISA using anti-pili (which mainly recognizes PilE, see *Experimental procedures*). The histogram shows the relative PilE expression levels of bacterial strains, OD_450_ value of the wild-type strain was set as 1.0. The error bars in C and D represent standard error of mean (SEM) from triplicate experiments. Statistically significant difference is indicated with single asterisk (*P*<0.05) or triple asterisks (*P*<0.001). E. Level of *pilE* mRNA were compared by quantitative RT-PCR. The histogram shows relative *pilE* mRNA levels normalized to the housekeeping genes, NMC0139 (50S ribosomal protein *rplP;* left three bars) or NMC0129 (30S ribosomal protein *rpsJ;* right three bars), and calculated in arbitrary units set to a value of 1 for the wild-type strain. The experiments were repeated using samples generated from two separate cDNA synthesises. Also, cDNA synthesized from a separate RNA preparation gave similar results (data not shown).

To investigate the function of NafA in *Neisseria* infection, we generated a NafA deficient mutant, ΔNafA, by replacing *nafA* with a kanamycin resistance cassette. A complemented strain, ΔNafA/NafA, was also constructed by introducing a copy of the *nafA* gene into a noncoding genomic region of the ΔNafA strain (see Experimental Procedures). The mutant and the complemented strain showed similar growth rate and colony morphology when compared to the wild-type strain (data not shown). Western blot analysis of outer membrane protein fractions (OMP) and whole cell lysates confirmed the absence of NafA in the mutant and showed that the complemented strain had regained NafA expression (Fig. 3A and B). To verify that the mutant was unaffected in the expression of major virulence factors, we analyzed whole cell lysates by western blot. There were no detectable differences in expression of Opa proteins, PilC, PilQ, or PilT when comparing the wild-type, the ΔNafA, and the complemented strains (Fig. 3B). However, the normalized intensity of the PilE-reactive band was 1.5-fold higher in the ΔNafA strain than that in the wild-type strain (Fig. 3C). Following this western blot, the amounts of pili on the bacterial surface were investigated by whole bacterial ELISA with an anti-pili antibody. We confirmed our PilE western blot result by finding that significantly more pili were expressed on the surface of the ΔNafA strain than on the wild-type strain (Fig. 3D). However, there were no differences in *pilE* gene mRNA amounts between the wild-type strain and the ΔNafA strain, as measured by a quantitative PCR assay (Fig. 3E).

**Figure 4:**
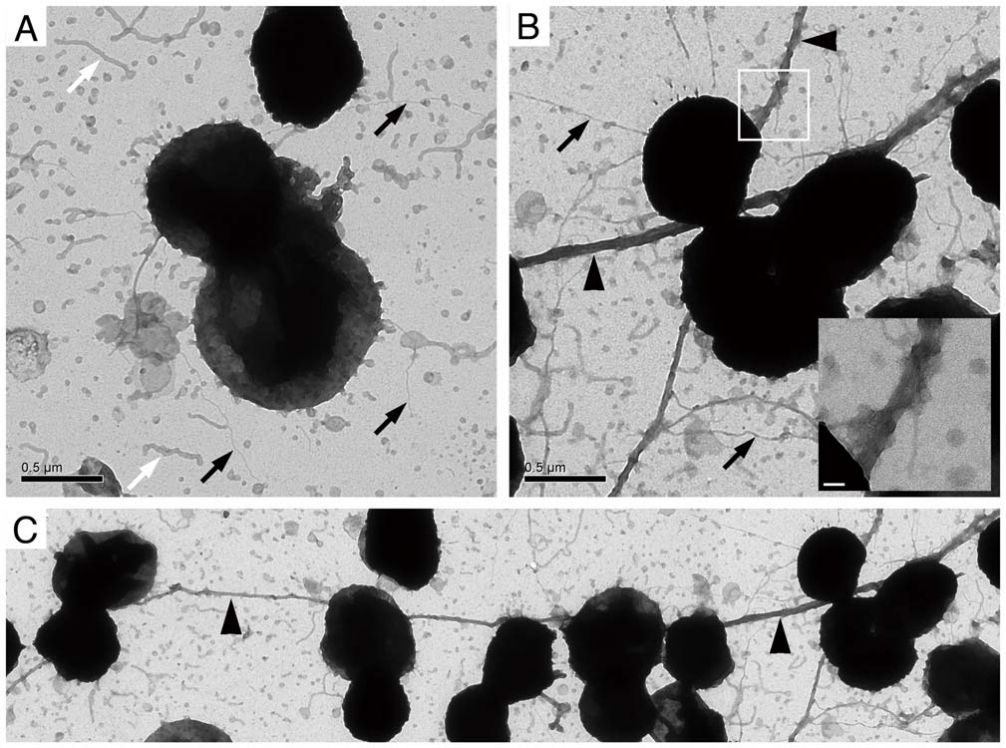
NafA regulates bacterial piliation. Piliation of the wild-type strain (A) and the ΔNafA strain (B, C) was analyzed by transmission electron microscope after negative staining. White arrows mark membrane blebs and black arrows mark pili. Arrowheads in panel B and C indicate thick bundled pilus structures. The inset in panel B shows a higher magnification of the boxed area, which displays the bundled pili. An overview of the ΔNafA strain piliation is shown in panel C. Black scale bars show 0.5 µm and white scale bars 50 nm.

Because pili play an important role in bacterial microcolony formation, transmission electron microscopy was used to evaluate whether NafA had any influence on cell or pilus morphology of *N. meningitidis*. As shown in Fig. 4, the ΔNafA strain expressed thick bundles of pili with an average length exceeding 20 µm (Fig. 4B and C, black arrowheads), whereas the wild-type strain lacked extended pilus bundles (Fig. 4A). We looked at more than 20 areas for each strain. Each area contained 10 – 30 diplococci. The thick bundles of pili, which associated with more than 10 diplococci as shown in Fig. 4C, was found in all areas of the ΔNafA strain. In contrast, we found no thick bundles of pili in the wild-type specimen. These results suggest that NafA controls piliation properties at the post-transcriptional level by preventing excessive bundling of pili.

### NafA prevents massive aggregation during meningococcal attachment to host cells

**Figure 5:**
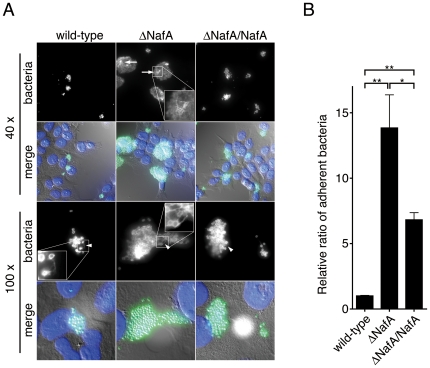
NafA negatively affects meningococcal attachment to host cells. A. FaDu cells were infected with the wild-type strain, the ΔNafA strain, or the ΔNafA/NafA strain for 2 h (moi = 200). After washing and fixation, the cells were stained with an anti-*N. meningitidis* pili antibody, which also stains the whole bacterial surface. Cell nuclei were stained with DAPI. The images were acquired with 40 x (upper two rows) and 100 x (lower two rows) objectives. Signals from anti-pili channel (“bacteria”) and merged images with DAPI stained cells (“merge”) are shown. Arrows and arrowheads indicate the pilus network-like structures and pilus fibres, respectively. B. FaDu cells at 2.5×10^4^ cells/well in 24-well plates were infected with 5×10^6^ bacteria (moi = 200). After 2 h of infection, 4±0.07×10^4^ bacteria were recovered from wells infected with the wild-type strain, *i.e.* about 1% of input bacteria were adhered to host cells. Adhesion of the bacterial strains in panel A was quantified and expressed as ratios relative to the wild-type strain, which was defined as 1. The error bars represent SEM of triplicate experiments. Statistically significant difference in adherence is indicated with two asterisks (*P*<0.001) or one asterisk (*P*<0.05).

Colonization of epithelial cell surfaces is an essential early step in meningococcal infection processes and pili play a crucial role during the initial bacterial attachment to host cells. To examine whether NafA affects pilus-mediated attachment, FaDu cells were infected with the wild-type strain or the ΔNafA strain for 2 h. The infected cells were then fixed and stained by an anti-*N. meningitidis*-pilus antibody, which also stains the whole *N. meningitidis* cell in immunofluorescence. When compared to the wild-type strain, the ΔNafA strain formed much larger microcolonies on the host cell surface accompanied by an increase in overall fluorescence intensity (Fig. 5A). Furthermore, network-like pilus structures were often seen in microcolonies of the ΔNafA strain, indicating the formation of thick bundled pili (Fig. 5A, arrows, inset in top row). The microcolonies formed by the complemented strain (ΔNafA/NafA) showed a pattern of staining comparable to the wild-type strain (Fig. 5A, right column). The bacterial adhesion was further analyzed by counting colony-forming units (CFU) after removal of unbound bacteria. As expected, significantly higher numbers of the ΔNafA strain were recovered from host cells compared to the wild-type strain. The enhanced attachment displayed by the ΔNafA strain was attenuated in the complemented strain (Fig. 5B). It has been shown that the antigenic variation of pilin strongly affects adhesiveness of *N. meningitidis* to human epithelial cells [26], [27]. These reports led us to examine the *pilE* gene sequences in the wild-type strain (FAM20), the ΔNafA strain, and ΔNafA/NafA strain. However, there were no sequence differences in *pilE* genes among the strains, showing that the high adhesiveness of the ΔNafA strain was not due to antigenic variation in *pilE*.

These results suggest that NafA negatively regulates bacteria-host cell interactions by reducing microcolony formation and excessive pilus aggregation, in a *pilE* sequence independent manner.

### NafA-mediated anti-aggregation is host cell independent

**Figure 6:**
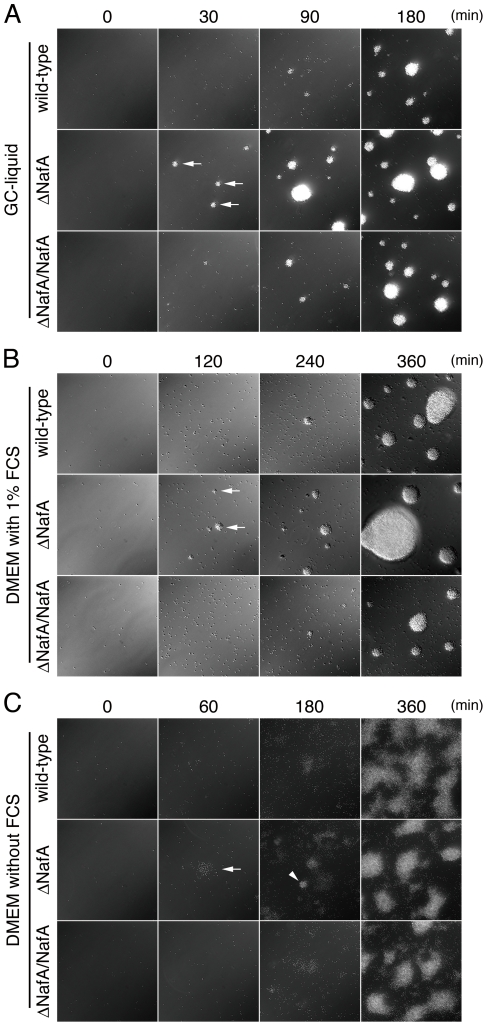
NafA regulates bacterial aggregation. The wild-type strain, the ΔNafA strain, or the ΔNafA/NafA strain was suspended in GC-liquid (A), DMEM with 1% FCS (B), and DMEM without FCS (C). The formation of bacterial aggregates was analyzed at indicated time points using a living-cell microscope at 37°C in an atmosphere of 5% CO_2_. Arrows in panel A and B indicate small bacterial aggregates. The arrow and arrowhead in panel C show a loose and a spherical bacterial aggregate, respectively.

**Figure 7:**
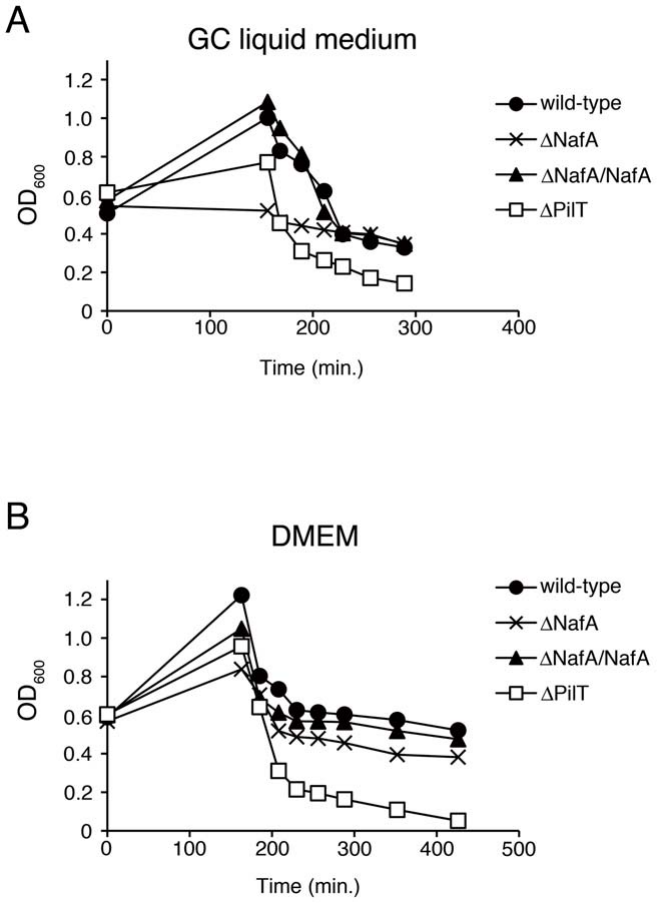
Quantification of bacterial aggregation. Aggregation was quantified by measuring the decrease in absorbance that occurs upon sedimentation of bacterial aggregates in GC liquid medium (A) or DMEM (B) under static conditions. Apparent differences in OD values (0–160 min) were due to aggregative properties, not due to growth rates.

To examine whether the enhanced microcolony formation of the ΔNafA strain was host cell-dependent, bacteria were grown in different liquid conditions, *i.e.* GC-liquid media, Dulbecco's modified Eagle's medium (DMEM) with and without 1% fetal calf serum (FCS). Bacterial aggregation was observed over a period of 6 h. After 30 min of incubation in GC-liquid or 120 min of incubation in DMEM containing FCS, small bacterial aggregates were observable for the ΔNafA strain (Fig. 6A and 6B, arrows), whilst most of the wild-type strain was still present as single diplococcus. When DMEM without FCS was applied, the ΔNafA strain started to form loose aggregates after 60 min of incubation (Fig. 6C, arrow) and spherical bacterial aggregates could be detected after 180 min of incubation (Fig. 6C, arrowhead). The wild-type strain was unable to form spherical aggregates even after 360 min of incubation in this medium. The complemented strain (ΔNafA/NafA) showed a capacity to form aggregate comparable with the wild-type strain under all conditions studied. To quantify the bacterial aggregation, we also performed a previously described sedimentation assay [7]. Strains were cultured in either GC liquid medium or DMEM for 150 min with shaking. The cultures were then left to sediment at room temperature without shaking. OD_600_ values of the ΔNafA culture supernatant were significantly lower than that of the wild-type strain from the 150 to 210 min time points of GC liquid medium and from the 150 to 420 min time points in DMEM without FCS, representative assay are shown in Fig. 7. These results suggest that NafA negatively controls the formation of bacterial aggregates also in the absence of host cells.

### NafA enhances bacterial virulence and survival in blood

**Figure 8:**
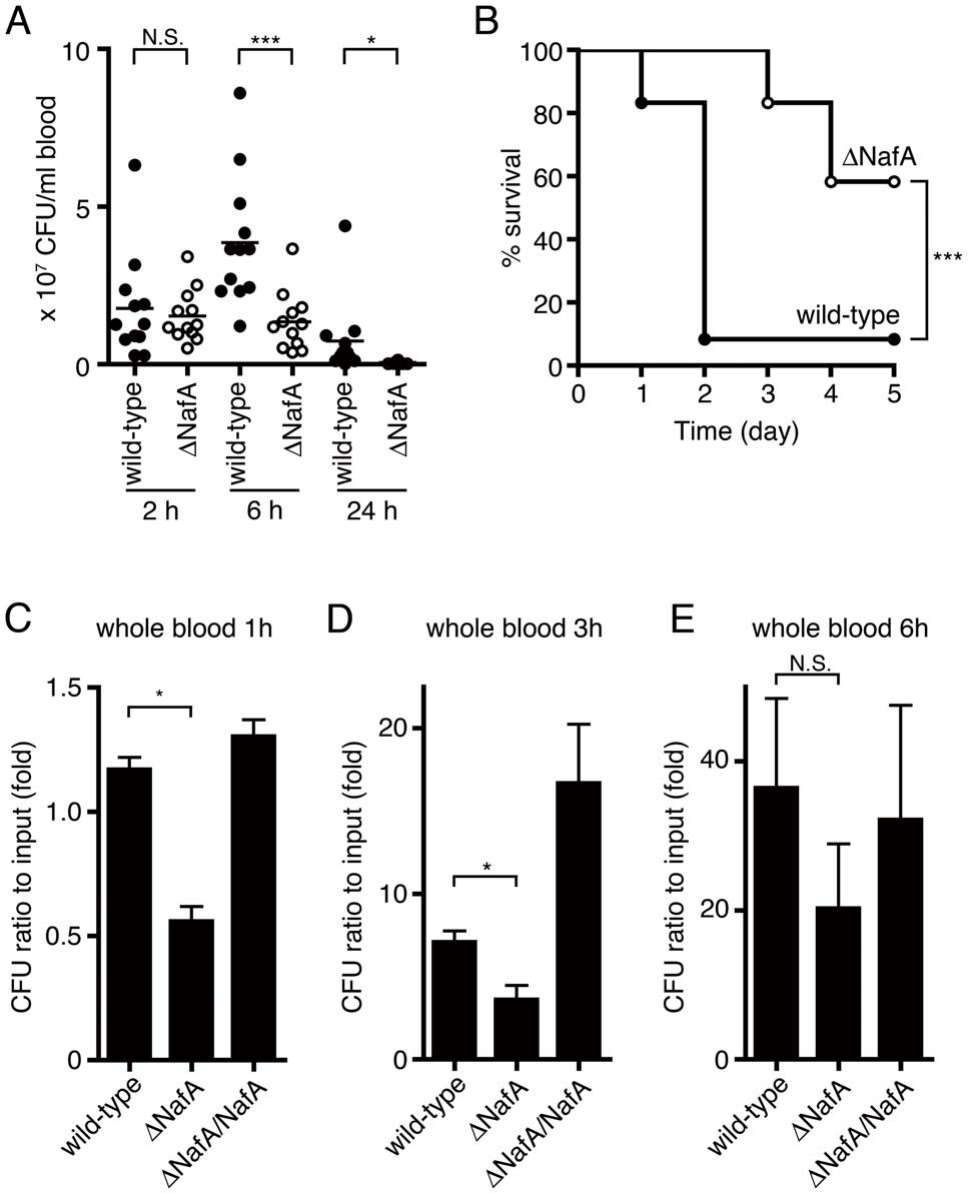
The ΔNafA strain displayed attenuated virulence during systemic infection. Human CD46 transgenic mice were infected i.p. with the wild-type strain (closed circles) or the ΔNafA strain (open circles) at a dose of 1×10^8^ CFU/mouse. A. Bacterial counts in blood (CFU/ml) were determined at 2 h, 6 h, and 24 h after challenge with each strain. Challenge with the ΔNafA strain resulted in lower levels of bacteremia than the wild-type strain at 6 h and 24 h post-infection. Statistically significant lower bacteremia compared to the wild type strain at each time point is indicated with an asterisk (*P<*0.05) or three asterisks (*P*<0.001). B. Survival rate of mice (n = 12 per group) after i.p. infection with the wild-type strain or the ΔNafA strain. The overall survival rates of the mice infected with the mutant were significantly lower (*P*<0.001) than those of the wild-type strain. C–E. Bacterial survival in whole blood collected from the human CD46 transgenic mice. Around 100 CFU bacteria were mixed with 10 µl whole blood at a 1∶1 volume ratio and incubated at 37°C in a 5% CO_2_ atmosphere for 1 h (C), 3 h (D), and 6 h (E). Bacteria were spread on GC agar plates and surviving bacteria were counted after overnight incubation. The data are presented as means ± S.E. of three independent experiments done in triplicate. Asterisk indicates significant difference (*P*<0.05). N.S. represents no significant difference.

Sepsis and meningitis are the major clinical features of meningococcal disease. To examine the impact of NafA during *N. meningitidis* disease *in vivo,* we challenged CD46 transgenic mice intraperitoneally (i.p.) with the ΔNafA and the wild-type strains. Blood samples were collected at indicated time points to monitor levels of bacteremia. At 2 h post infection, there was no significant difference in bacteremia levels between the wild-type strain and the ΔNafA strain (Fig. 8A), indicating that the mutant retained an ability to invade and survive in blood. At 6 h and 24 h post infection, the bacterial blood counts in mice challenged with the ΔNafA strain were significantly lower than in mice infected with the wild-type strain (Fig. 8A). The ΔNafA strain was also attenuated in its ability to cause lethal disease when compared to the wild-type strain. At 2 days post-infection nearly all mice (11 out of 12) challenged with the wild-type strain had a lethal outcome, while around 60% (7 out of 12 mice) of the mice infected with the mutant survived (Fig. 8B). These results suggest that expression of NafA contributes to bacterial survival in blood and consequently facilitates the development of lethal sepsis in the host. To confirm this hypothesis, the role of NafA in an *ex vivo* whole blood was investigated. Suspensions of the wild-type, the ΔNafA, and the ΔNafA/NafA strains, containing equal amounts of CFU, were incubated for 1 h, 3 h, or 6 h in whole blood collected from CD46 transgenic mice. Fig. 8C-E shows a 50% reduction in viable counts of the ΔNafA mutant when compared to the wild-type at the 1 h and 3 h time points. No difference could be detected between the complemented strain and the wild-type strain. To evaluate whether the ΔNafA strain in more sensitive to complement-mediated killing, bacterial strains were grown in 50% of serum collected from CD46 transgenic mice and incubated for 1 h. However, we did not observe any growth differences between the wild-type and the ΔNafA strain under these conditions (data not shown). Taken together, these results suggest that NafA, which is not involved in the protection from complement attack, facilitates bacterial survival in the bloodstream and contributes to meningococcal virulence.

## Discussion

The NafA (NMB0995) protein was first identified as an antigen with the capacity to induce bactericidal antibodies in mice [24]. Upon interaction with epithelial cells, expression of NafA increased and the protein could be detected at the bacterial surface, indicating that NafA might play an important role in the bacteria-host cell interaction. In this study, we investigated the impact of this protein on bacterial pathogenesis by using an isogenic NafA-deficient mutant strain (ΔNafA). We demonstrate that NafA negatively regulates pilus expression and plays a determining role in bacterial aggregation and disease development.

In order to generate a ΔNafA strain, we applied a MultiSite Gateway Three-Fragment Vector Construction system. This system can also be used to express exogenous genes in *Neisseria,* as demonstrated by the strategy used for construction of the complemented strain (ΔNafA/NafA). For the complementation of the *nafA* mutation, we constructed the plasmids pDONR P4-P1R 5-porA-pro and pDONR P2R-P3 CmR-3 to facilitate introduction of exogenous gene into a non-coding chromosomal region (Fig. S1 and S2). These two plasmids are universally useful to express genes under the *porA* promoter. We were also successful in expressing other genes, such as *egfp* derived from pEGFP-C1, in *N. meningitidis* FAM20, and it was possible to modulate gene expression by introducing a consensus ribosomal binding sequence (Fig. S3). It is also possible to exchange the *porA* promoter, used in this study, with any other promoter without major effort.

The levels of PilE and pili in the ΔNafA strain, as determined by western blot and whole-cell ELISA, were higher than that in the wild-type strain (Fig. 3). Transmission electron microscopy further showed that the ΔNafA strain formed thick bundles of pili, which linked several bacterial cells together (Fig. 4). These results demonstrate that NafA negatively contributes to bacterial adhesion (Fig. 5) and aggregation (Fig. 6 and 7), possibly by preventing excess pilus bundling. The difference in pili amounts on bacterial surfaces between the wild-type strain and the ΔNafA strain was unexpectedly little in the whole-cell ELISA (Fig. 3D). The reason could be speculated that the thick bundled pili of ΔNafA strain can be physically washed out more easily than wild-type pili during ELISA experiment.

The closest non-*Neisseriaceae* family orthologue of NafA is a *H. influenzae* hypothetical protein (locus tag: CGSHi3655_03561), sharing less than 60% identity to NafA, and it would be of great interest to find out if the anti-aggregative property of NafA is *Neisseria*-specific. The ATPase PilT is the sole component directly responsible for depolymerisation (*i.e.* retraction) of the pilus filament. PilT deficient mutants of *Neisseria* are hyperpiliated, hyperaggregative and display increased adherence to the host cell. However, PilT mutants display irregularly shaped aggregates, are non-motile, and lack natural competence [10], [28], [29], [30]. The ΔNafA strain forms spherical aggregates (Fig. 6) and retains natural transformation competence, as shown by the creation of the ΔNafA/NafA complemented strain. Furthermore, the ΔNafA strain displayed normal PilT expression (Fig. 3B) and motility (data not shown), indicating that NafA regulates bacterial piliation and auto-aggregation through a distinct mechanism.

The induction of NafA could only be detected by fluorescence microscopy at 6 hours post infection, but western blots of outer membrane fractions indicate that low amounts of NafA are constitutively expressed at the bacterial surface (Fig 3A). NafA lacks a typical signal peptide sequence, but contains a predicted transmembrane domain between amino acid 104–126 at the C-terminal part (Fig. 1B), as analyzed by the TMpred program (http://www.ch.embnet.org/software/TMPRED_form.html). The detailed molecular mechanisms of NafA translocation to the bacterial surface remain to be elucidated. The reason for the finding that the complemented strain still shows higher adhesiveness than the wild-type strain is unclear. The *nafA* gene in the complemented strain is controlled under *porA* promoter, and is integrated into the noncoding region that is far from the original locus. These situations might affect the efficient transportation of NafA in the complemented strain.

Survival of bacteria in blood is a prerequisite for meningococcal disease. Several mechanisms are used by *N. meningitidis* to enhance its survival in the host bloodstream, including expression of capsule [31] and certain outer membrane proteins [32]. We have earlier developed an animal model of *N. meningitidis* infection using human CD46 transgenic mice [33], [34]. Although the role of CD46 in *Neisseria* infection is still not absolutely clear [35], [36], the model is working [37] and is today to our knowledge the best available *in vivo* meningococcal infection model system. Bacterial loads in the circulation system 2 h post infection suggested that NafA deficiency had no influence on the traversal of bacteria from peritonea to the bloodstream. However, infection with the ΔNafA strain resulted in lower levels of bacteremia at late time points when compared to the wild-type strain. This led us to examine if NafA conferred resistance to whole blood mediated killing. When incubated with mouse whole blood, the wild-type strain showed increased survival when compared to the ΔNafA strain (Fig. 8C and 8D). However, both strains grew at a comparable level in mouse serum indicating that the anti-aggregation function of NafA could augment bacterial spreading and escape from phagocyte-mediated bacterial killing. It has been described that post-transcriptional modification of pili can affect bacterial opsonisation and by this mechanism enhance bacterial uptake by human phagocytes [38]. Thus, NafA could play a similar, indirect, role in protecting bacteria from phagocytosis. It is worth noting that the ΔNafA strain forms microcolonies much quicker than the wild-type strain, even when we filtered the bacterial suspensions before animal challenge. Therefore, the bacterial number of the ΔNafA strain we challenged to the mice (Fig. 8A and 8B) might be higher than that of the wild-type strain. However, mice challenged with the ΔNafA strain displayed better survival suggesting that negative modulation of bacterial aggregation is important for bacterial survival during systemic infection.

This study reveals an important role of NafA in bacteria-host cell interaction and suggests that bacterial microcolony formation is regulated by both positive, such as PilX, and negative, such as NafA, aggregation factors.

In conclusion, we find that in order to separate aggregated bacteria formed during initial attachment, NafA is expressed at the bacterial surface, and that appropriate separation of bacterial cells seems to enhance bacterial survival in blood. We further hypothesize that NafA suppresses the excess accumulation of extracellular pilin and that in the absence of NafA, pilin secretion increased, protecting extracellular pilin from degradation. The increased secretion of pilin causes a lowered intracellular concentration of pilE, which in turn does not seem to be subjected to positive auto-regulation, as has been hypothesized for *N. gonorrhoeae* MS11 [39].

## Materials and Methods

### Ethics Statement

Mice experiments described in the present study were conducted at the animal facility of Stockholm University. Animal care and experiments were conducted adhering to the Swedish guidelines for animal husbandry. All protocols were approved by the Swedish Ethical Committee on Animal Experiments (Approval ID: N380/08).

### Bacterial strains, cell culture, and growth conditions

The wild-type strain used in this study was *N. meningitidis* serogroup C FAM20 [40]. PilT-deficient mutant (ΔPilT) were derived from FAM20 [29]. *Neisseria* strains were grown on GC agar plates [41] or in GC liquid media containing 1% Kellogg's supplement [42] at 37°C in a 5% CO_2_ atmosphere. *E*. *coli* DH10B (Invitrogen) was used for cloning. For selection of *N. meningitidis* transformants, kanamycin and chloramphenicol was added to culture media at the concentration of 100 and 2 µg/ml, respectively. FaDu cells (ATCC HTB-43) were maintained in DMEM (Invitrogen) supplemented with 10% heat-inactivated FCS. Unless stated otherwise, all experiments were performed using 80% confluent cells cultivated in 24-well cell culture plate (105 cells/well).

### Generation of anti-NafA antibodies

To prepare the anti-NafA antibodies, the peptides N, M, and C (Fig. 1B) corresponding to the amino acid residues 10 – 31 (CETAPEAAKARVEAVLQNNGFIP), 97 – 113 (CQSVKAARALAAGEFDDA), and 128 – 144 (CKKGAVSDDELKAFFDAG) of NafA were conjugated separately with hemocyanin from keyhole limpets (Sigma). A cysteine residue was added at the N-terminal of each peptide in order to cross-link the carrier protein. The cross-linked peptides were used to immunize rabbits. In order to obtain specific immunoglobulin fractions, all antisera obtained were absorbed once with the peptide immobilized on the epoxy-activated sepharose 6B (GE Healthcare).

### Adherence assays

Bacterial colonies grown overnight on GC agar plates were suspended into DMEM cell culture media and filtered through a 5 µm-pore filter to obtain separated bacteria. The FaDu cells were infected with bacteria at a multiplicity of infection (moi) of 200. After a 2 h incubation at 37°C in a 5% CO_2_ atmosphere, unbound bacteria were washed away with phosphate-buffered saline (PBS), the cells were then lysed with 1% saponin in GC-liquid media for 5 min. Lysates were plated onto GC agar plates, incubated overnight and CFU were counted. For immunofluorescence, FaDu cells were cultured on cover slides (In vitro diagnostics) and infected with bacterial strains for 2 h and 6 h at 37°C in a 5% CO_2_ atmosphere. After washing away unbounded bacteria, the cells were fixed with 3.7% formaldehyde, stained with an anti-*N. meningitidis* pili antibody or anti-NafA antibody. The secondary antibody applied was an anti-rabbit IgG-Alexa 488 (Invitrogen) and DNA was visualized by DAPI staining. Images were captured using an inverted Zeiss Axio Observer microscope. Each experiment was independently repeated for at least three times.

### Generation of the NafA mutant

Primers used in this study are shown in Table 1. The MultiSite Gateway Three-Fragment Vector Construction Kit together with the adaptor PCR method of the Gateway cloning system (Invitrogen) was used to construct the NafA deficient mutant. A 574 bp upstream region of the *nafA* gene was amplified from FAM20 with the primers B4-NMC0982 and B1R-NMC0982. The resulting PCR product was cloned into pDONR P4-P1R to obtain pDONR P4-P1R Δ*nafA*. A 555 bp downstream region of the *nafA* was amplified with the primers B2R-NMC0982 and B3-NMC0982. The resulting PCR product was cloned into pDONR P2R-P3 to obtain pDONR P2R-P3 Δ*nafA*. A 1075 bp DNA fragment containing a kanamycin resistance gene was amplified from plasmid pDONR P4-P1R with the primers B1-KmR and B2-KmR. The resulting PCR product was cloned into pDONR 201 to obtain pDONR KmR. Above constructed plasmids pDONR P4-P1R Δ*nafA*, pDONR KmR, and pDONR P2R-P3 Δ*nafA* were mixed with pDEST R4-R3 (Invitrogen) and LR Clonase Plus Enzyme in order to connect all cloned fragments, *i.e.* the upstream region of *nafA* gene, the kanamycin resistance gene, and the downstream region of *nafA* gene, in order. The resulting plasmid designated pDEST-Δ*nafA*-KmR was inserted into the genome of FAM20 by homologous allelic replacement after transformation [43]. The replacement of the *nafA* by the kanamycin resistance gene was confirmed by PCR and western blot. The *nafA-*deficient strain was designated ΔNafA.

**Table 1:** Primers used for cloning.

Name	Sequence of primer pairs
B4-NMC0982	5′-ATAGAAAAGTTGGCAAGGCGAGGTAACGCCGTACTGG-3′
B1R-NMC0982	5′-TGTACAAACTTGTTAAATGCCGTCTGAACATTTTTCAGAC-3′
B2R-NMC0982	5′-TGTACAAAGTGGATGGAAATCAACCCCGAATTGCAGGCTTACG-3′
B3-NMC0982	5′-ATAATAAAGTTGTTTGCCTTGCAGGATGTCGGCAAGG-3′
B1-KmR	5′-AAAAAGCAGGCTGTATTAGTGACCTGTAGAATTCGAGC-3′
B2-KmR	5′-AGAAAGCTGGGTTTAGAAAAACTCATCGAGCATCAAATG-3′
B4-DUS-5	5′-ATAGAAAAGTTG*ATGCCGTCTGAA*AATTAAGTTAGAATTATCCC-3′
5-porA-pro-L	5′-CGCGGCACTTTAAAATCCAAAATCATACTGCC-3′
5-porA-pro-U	5′-GATTTTGGATTTTAAAGTGCCGCGTGTGTTTTTTTATGGC-3′
B1R-porA-pro	5′-TGTACAAACTTGATCTGCTTCCTTTTGTAAATTTGATAAAAACC-3′
B2R-Cm-U	5′-TGTACAAAGTGGCTAAGTTGGCAGCATCACCC-3′
Cm-3-L	5′-TGGTATCCGACCATATTGACATCATATATGCC-3′
Cm-3-U	5′-TGATGTCAATATGGTCGGATACCAATCTGACG-3′
B3-3	5′-ATAATAAAGTTGCTAACAGAAAACTCTACTCC-3′
B1-NMC0982-comp	5′-AAAAAGCAGGCTTCGGCGCAAAGGGCAACAGCC-3′
B2-NMC0982-comp	5′-AGAAAGCTGGGTTCAGGCGTAAGCCTGCAATTCGGGG-3′
pilE-U	5′-TATTCCGACAACGGCACATTCCC-3′
pilE-L	5′- CCTTCAACCTTAACCGATGCCA-3′
rpsJ-U	5′-TTGGAAATCCGCACCCACTT-3′
rpsJ-L	5′-TACATCAACACCGGCCGACAAA-3′
rplP-U	5′-GTGGCGGTAAAGGTAACGTGGAAT-3′
rplP-L	5′-TCGAATGCTTCACGAGCCAGTT-3′

* The DNA uptake sequence is marked in italic.

The overlapping homologous sequence for combined PCR is underlined.

### Complementation of ΔNafA

The ΔNafA strain was complemented by introducing a wild-type gene copy into a non-coding genomic region between NMC0075 and NMC0080 by homologous allelic replacement [43]. The MultiSite Gateway Three-Fragment Vector Construction Kit and the adaptor PCR were applied as described above. A 502 bp fragment containing whole NMC0075 ORF (upstream region of the insertion) was amplified from FAM20 with the primers B4-DUS-5, which contains a DNA uptake sequence [44], and 5-porA-pro-L. A 324 bp fragment containing the transcriptional promoter for *porA* gene was amplified with the primers 5-porA-pro-U and B1R-porA-pro. The NMC0075 fragment and the *porA* promoter fragment were connected by PCR done in two steps, ten cycles without any primers followed by the adapter PCR cycle with B4 and B1R universal primers (Invitrogen). The combined fragment was cloned into pDONR P4-P1R, which resulted in plasmid pDONR P4-P1R 5-porA-pro (Fig. S1). An 1118 bp fragment containing the chloramphenicol resistance gene was amplified by PCR with the primers B2R-Cm-U and Cm-3-L using pDONR 201 plasmid DNA as the template. A 515 bp fragment containing the upstream region of NMC0080 (downstream region of the insertion) was amplified from FAM20 with the primers Cm-3-U and B3-3. The chloramphenicol resistance gene fragment and the NMC0080 region fragment were connected by PCR using B2R and B3 universal primers (Invitrogen) as described above. This combined fragment was cloned into pDONR P2R-P3, which resulted in plasmid pDONR P2R-P3 CmR-3 (Fig. S1). An 858 bp DNA fragment containing full-length *nafA* gene was amplified with the primers B1-NMC0982-comp and B2-NMC0982-comp. The resulting PCR product was cloned into pDONR 201 (Invitrogen) to obtain pDONR *nafA* (Fig. S2). The three plasmids obtained, such as pDONR P4-P1R 5-porA-pro, pDONR *nafA*, and pDONR P2R-P3 CmR-3 were mixed with pDEST R4-R3 together with LR Clonase Plus Enzyme to combine the NMC0075 region, *porA* promoter fragment, *nafA* gene, the chloramphenicol resistance gene, and NMC0080 region in order. The resulting plasmid, designated pDEST-*nafA*-comp (Fig. S2), was then introduced into *N. meningitidis* ΔNafA genome by transformation. The correct integration of *nafA* gene was confirmed by PCR and western blot. The complemented strain was designated ΔNafA/NafA.

### Western blot analysis

Expression of NafA and other virulence related factors were detected by western blot. Anti-Opa mouse monoclonal antibodies [37], and rabbit polyclonal anti-PilT [29], PilQ [45], PilC, Pili [37] antibodies were applied as primary antibody. Horseradish peroxidase (HRP) conjugated anti-mouse or rabbit IgG (Bio-Rad) was used as secondary antibody. ECL (PerkinElmer) and a CCD camera (Fujifilm) were used to detect the signals. Signal intensities of bands were measured by ImageJ (http://rsbweb.nih.gov/ij/) software.

To prepare whole bacterial protein extracts, colonies grown overnight on GC plates were suspended in PBS and adjusted to OD_600_ 1.0. One ml of the bacterial suspension was mixed with 100 µl of 100% trichloroacetic acid followed by 15 min incubation on ice. After 5 min centrifugation at 20,000 x *g,* the pellet was dissolved in SDS-PAGE sample buffer. Outer membrane protein fractions were obtained according to a protocol described previously [46]. In brief, bacteria suspended in LiCl buffer (0.2 M LiCl, 0.1 M sodium acetate, pH 5.8) were vigorously shaken with 4 mm diameter glass beads. After centrifugation, supernatants were subjected to ultracentrifugation at 100,000 x *g* for 2 h. The pellets were then dissolved in the SDS-PAGE sample buffer. OMP analysis and western blots were repeated for at least three times using the samples prepared independently.

### Whole cell ELISA

The ELISA was performed as described previously [29]. Wells of 96-well microtiter plates were coated with 50 µl of bacteria suspension in PBS (OD_600_ = 0.01) for 2 h at room temperature, blocked with 5% bovine serum albumin for 2 h at room temperature. After 2 h incubation at room temperature with anti-pili antibody used in the western blot analyses of this study, HRP-conjugated anti-rabbit antibody was added as secondary antibody. The plates were then incubated for 2 h at room temperature, and plate-bound peroxidase was detected using TMB (3, 3′, 5, 5′-tetramethylbenzidine) and stop solution. Finally OD450 of each well was measured using a plate reader.

### mRNA analysis by quantitative PCR

*Neisseria* strains were grown on GC plates for 18 hours. Total RNA was isolated using a modified protocol for the SV Total RNA Purification kit (Promega) including initial phenol/ethanol incubation on ice to stabilize the RNA and prevent degradation. RNA yield and quality was assessed using NanoDrop 8000. Template RNA (150 ng) from each strain was reverse-transcribed into cDNA with random hexamers using Superscript III First-Strand Synthesis (Invitrogen). The resulting cDNA (0.1 µl) was amplified using LightCycler® 480 SYBR Green I Master kit (Roche Diagnostics) together with *pilE* primers, NMC0129 (*rpsJ*; 30 S ribosomal protein) primers and NMC0139 (*rplP*; 50S ribosomal protein) in a LightCycler® 480 Real-Time PCR System (Roche Diagnostics). Primer pairs specific for *pilE* (pilE-U and pilE-L), NMC0129 (rpsJ-U and rpsJ-L) and NMC0139 (rplP-U and rplP-L) were used at a final concentration of 500 nM. PCR program was adapted from LightCycler® 480 SYBR Green I Master kit (Roche Diagnostics) with an annealing temperature of 60 °C. Data analysis was performed with the LightCycler® 480 Software 1.5 using the comparative cycle threshold method, in which target mRNA is normalized to the reference genes, NMC0129 and NMC0139, and calculated in arbitrary units set to a value of 1 for the wild-type strain. The specificity was checked by analyzing the melting curves.

### Electron microscopy

To analyze the structure of pili, we followed a previously described protocol [7]. In brief, bacteria suspended in PBS were adsorbed to formvar-coated grids for 5 min and fixed with 1% glutaraldehyde for 5 min. After washing twice with water, grids were incubated with 1% phosphotungstic acid for 2 min, air-dried and analyzed using a TECNAI G2 Sprint BioTWIN electron microscope (FEI).

### Quantification of the bacterial aggregative ability

We followed a previously described protocol [7]. Bacteria grown overnight on GC agar plates were resuspended GC liquid medium or DMEM, filtered on 5 µm filters to eliminate large aggregates and adjusted to an OD_600_ of 0.6 in 30 ml of the each medium. Bacteria were then grown for 150 min under constant agitation. Finally, these suspensions were left to sediment at room temperature without shaking, and the OD_600_ of the supernatant was measured at 20 min intervals.

### Mouse model of meningococcal infection

Five to seven weeks old human CD46 transgenic mice [33], [34], [47] were used as disease model. Bacteria grown overnight on GC agar plates were resuspended in PBS, filtered through a 5 µm-filter and the viable counts were determined at once. To study systemic disease, mice (n = 6 in each group) were challenged i.p. with 1×10^8^ CFU bacteria in 100 µl PBS. Mice were monitored for 7 days in survival studies. Bacteremia was determined by recovery of bacteria from blood samples taken from the tail vein at different time points post challenge. This experiment was repeated twice.

### Whole blood killing assay

Whole blood was taken from the orbital vein of the mice and heparinized. Bacterial colonies grown overnight on GC plates were suspended in DMEM. Bacterial suspensions containing 10^2^ CFU of *N. meningitidis* (10 µl) were mixed with 10 µl of blood and incubated at 37°C in a 5% CO_2_ atmosphere for indicated time periods. Bacterial suspensions were then plated on GC plates to enumerate viable bacteria. Assays were performed twice in triplicates and data show the ratio of the recovered bacteria compared to the initial input.

### Statistical analysis

The Student's *t*-test was used in quantification of band intensities (Fig. 3C), whole cell ELASA (Fig. 3D), adherence efficiency experiment (Fig. 5) and whole blood killing assay (Fig. 8C, 8D, and 8E). Bacterial counts in blood were analyzed using non-parametric Mann-Whitney test (Fig. 8A). Survival rates were assessed using the log rank test (Fig. 8B). Values of *P*<0.05 were considered significant.

## Supporting Information

Figure S1 Construction of pDONR P4-P1R 5-porA-pro and pDONR P2R-P3 CmR-3. A non-coding genomic region between NMC0075 and NMC0080 was selected for insertion of the complementary *nafA* gene by homologous allelic replacement. A 842 bp fragment containing a 502 bp upstream region and the *porA* promoter generated by combination PCR (See Experimental procedures) was cloned into pDONR P4-P1R to obtain pDONR P4-P1R 5-porA-pro. A DNA uptake sequence (DUS) was introduced at the 5′ end of this fragment. A 1649 bp fragment containing chloramphenicol resistance gene (Cm^R^) and a 515 bp downstream region obtained by combination PCR was cloned into pDONR P2R-P3 to produce pDONR P2R-P3 CmR-3.
(PDF)

Figure S2 Construction of plasmid pDEST-*nafA*-comp using a MultiSite Gateway strategy. A full-length *nafA* ORF was cloned into pDONR 201 to obtain pDONR *nafA* (See Experimental procedures). Multiple fragments carried by plasmids pDONR P4-P1R 5-porA-pro, pDONR *nafA* and pDONR P2R-P3 CmR-3 were assembled after mixing with the plasmid pDEST R4-R3 containing specific recombination sites and the LR clonase Plus enzyme according to the supplier's instruction. The resulting plasmid pDEST-*nafA*-comp containing upstream region (5′), *porA* promoter (P*_porA_*), *nafA* ORF, chloramphenicol resistance gene (Cm^R^), and downstream region (3′) was used to complement the ΔNafA mutation via transformation.
(PDF)

Figure S3 Construction of a universal expression vector for *N. meningitidis* and expression of the *egfp* gene in *N. meningitidis* FAM20. A. The *egfp* ORF was PCR amplified from the pEGFP-C1 plasmid (Clontech) with the primers B1-egfp (AAA AAG CAG GCT ATG GTG AGC AAG GGC GAG GAG CTG TTC) and B2-egfp (AGA AAG CTG GGT TTA TCT AGA TCC GGT GGA TCC CGG G). To enhance the gene translation, an RBS*-*fused *egfp* ORF was also amplified with the primers B1-RBS-egfp (AAA AAG CAG GCT AGG AGG ATC CT**A TG**G TGA GCA AGG GCG AGG AGC TGT TG) and B2-egfp. RBS is underlined and the start codon of *egfp* gene is in bold. These two *egfp* containing fragment were cloned into pDONR 201, resulting plasmids pDONR *egfp* and pDONR RBS-*egfp*, respectively. The *egfp* ORF was assembled with the upstream region containing the *porA* promoter and the downstream fragment containing Cm^R^ after mixing plasmids pDONR P4-P1R 5-porA-pro, pDONR *egfp* (or pDONR RBS-*egfp*), pDONR P2R-P3 CmR-3, and pDEST R4-R3 as described in Fig. S2. The resulting expression plasmid was designated as pDEST-*egfp* or pDEST-(RBS)-*egfp.* B. *egfp* gene expression detected by western blot analysis. Plasmid pDEST-*egfp* or pDEST-RBS-*egfp* was transformed into *E. coli* DH10B and the *N. meningitidis* strain FAM20. Bacterial cell lysates were prepared and EGFP was detected using an anti-GFP monoclonal antibody (Roche). Parental bacterial strains were used as negative control (control). EGFP signal intensities from bacteria containing the additional RBS (RBS-EGFP) were higher than those lacking the additional RBS (EGFP) in both *E. coli* and *N. meningitidis*.
(PDF)

Figure S4 Anti-NafA antibody prepared in present study is specific for NafA FaDu cells were infected with the wild-type strain (left column, identical photos in Fig. 2) or the ΔNafA strain for 6 h (right column). After washing away unbound bacteria, the cells were stained with anti-NafA peptide N antibody. Fluorescent (upper row) and Nomarski (lower row) images are shown. A 100 x objective lens was used. The inset shows higher magnification of the boxed area in the image.
(PDF)
