# Supplementary figures and images for: NafA Negatively Controls Neisseria meningitidis Piliation
Source: PLoS One. 2011 Jul 1;6(7):e21749. doi: 10.1371/journal.pone.0021749 (PMC3128610; doi:10.1371/journal.pone.0021749)

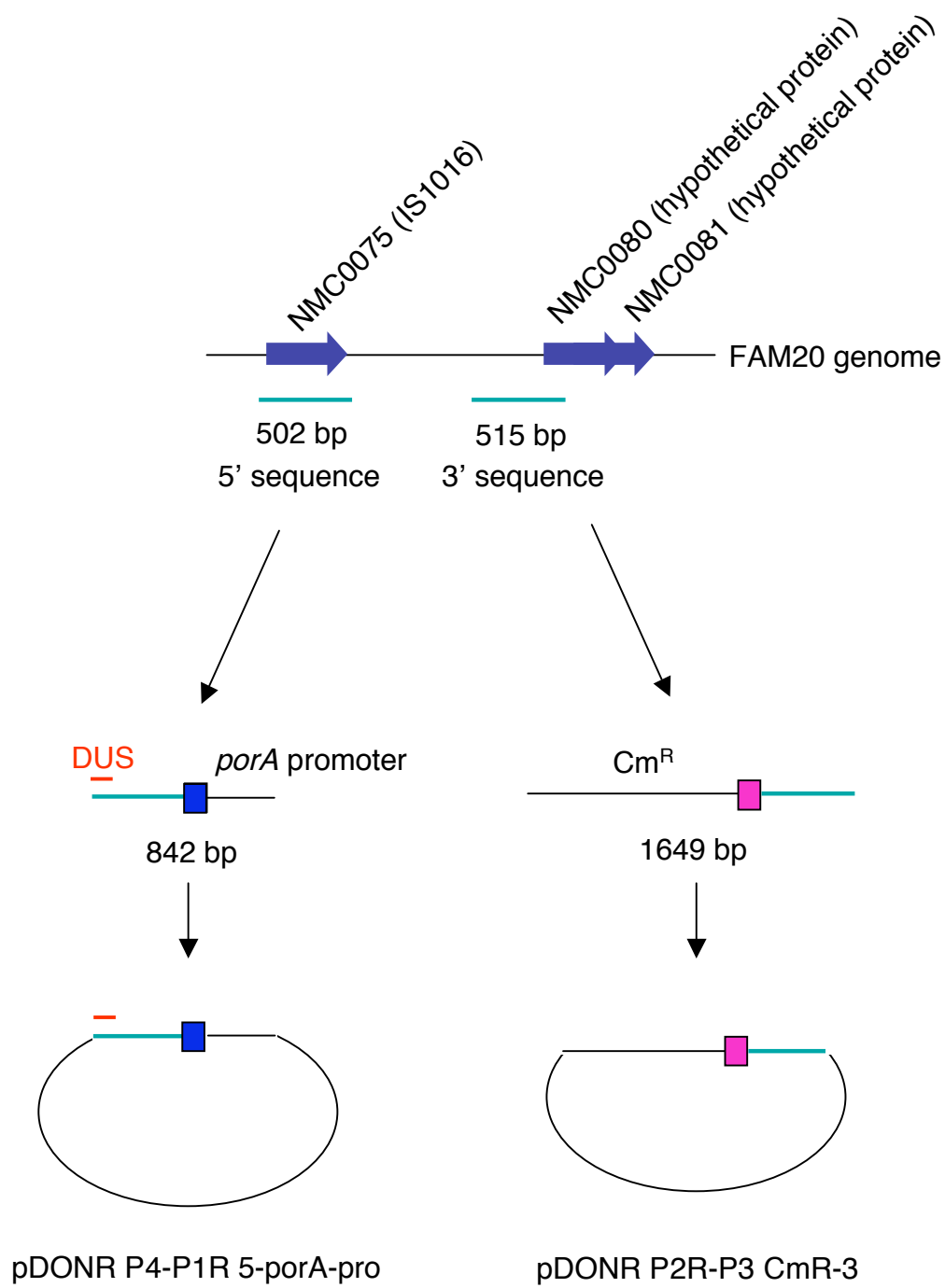

Fig. S1

Supplement: Figure S1 — Construction of pDONR P4-P1R 5-porA-pro and pDONR P2R-P3 CmR-3. A non-coding genomic region between NMC0075 and NMC0080 was selected for insertion of the complementary nafA gene by homologous allelic replacement. A 842 bp fragment containing a 502 bp upstream region and the porA promoter generated by combination PCR (See Experimental procedures) was cloned into pDONR P4-P1R to obtain pDONR P4-P1R 5-porA-pro. A DNA uptake sequence (DUS) was introduced at the 5′ end of this fragment. A 1649 bp fragment containing chloramphenicol resistance gene (CmR) and a 515 bp downstream region obtained by combination PCR was cloned into pDONR P2R-P3 to produce pDONR P2R-P3 CmR-3. (PDF) [file pone.0021749.s001.pdf]

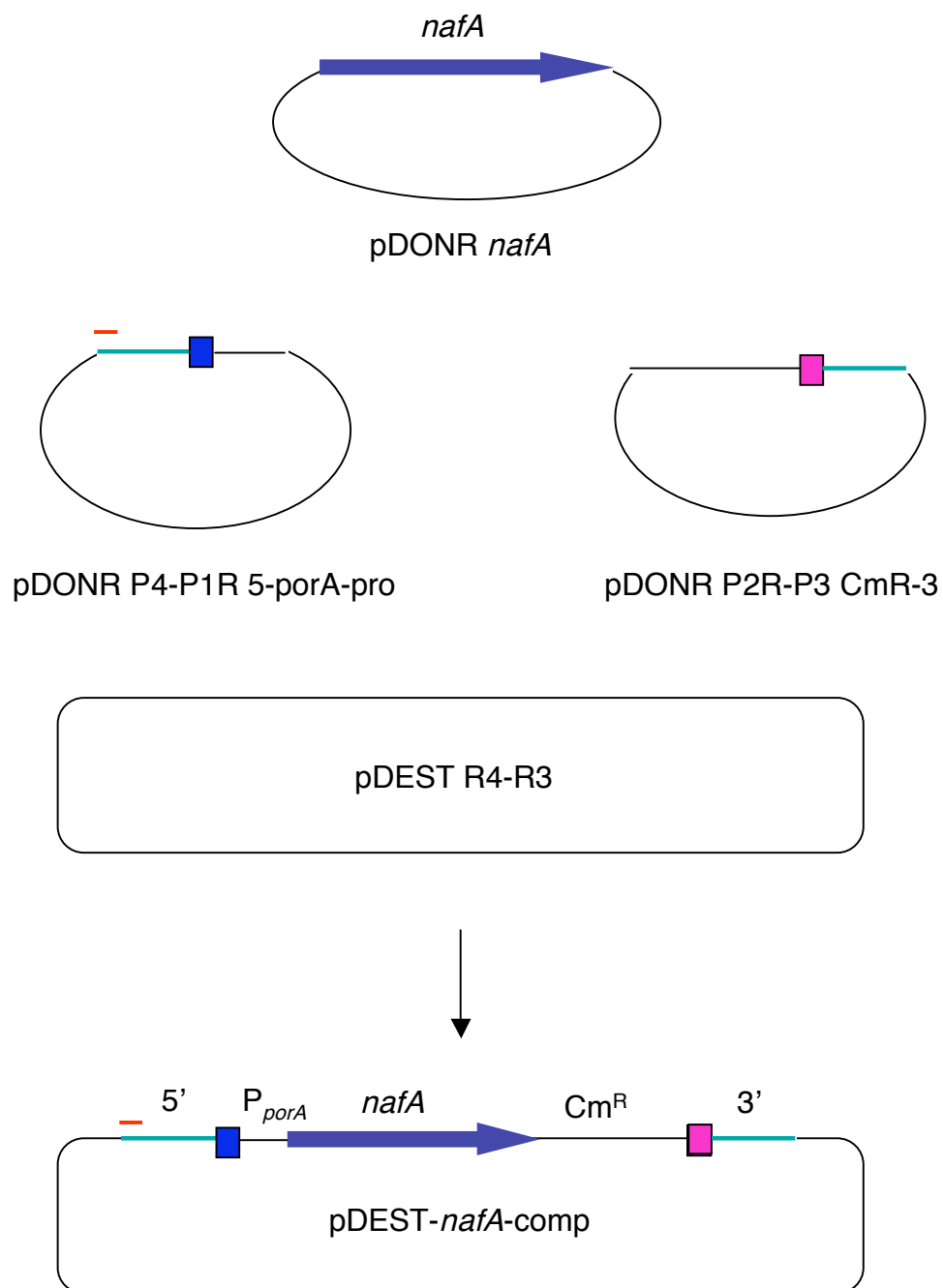

Fig. S2

Supplement: Figure S2 — Construction of plasmid pDEST-nafA-comp using a MultiSite Gateway strategy. A full-length nafA ORF was cloned into pDONR 201 to obtain pDONR nafA (See Experimental procedures). Multiple fragments carried by plasmids pDONR P4-P1R 5-porA-pro, pDONR nafA and pDONR P2R-P3 CmR-3 were assembled after mixing with the plasmid pDEST R4-R3 containing specific recombination sites and the LR clonase Plus enzyme according to the supplier's instruction. The resulting plasmid pDEST-nafA-comp containing upstream region (5′), porA promoter (PporA), nafA ORF, chloramphenicol resistance gene (CmR), and downstream region (3′) was used to complement the ΔNafA mutation via transformation. (PDF) [file pone.0021749.s002.pdf]

A

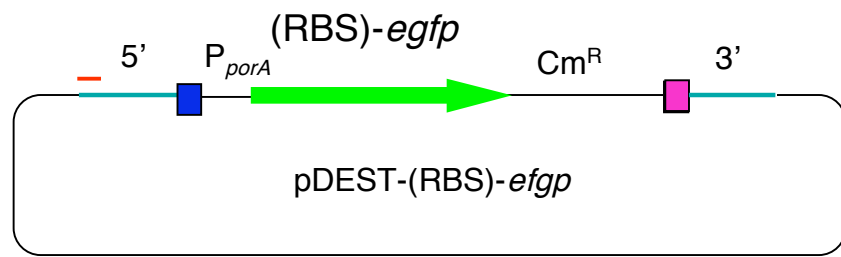

B

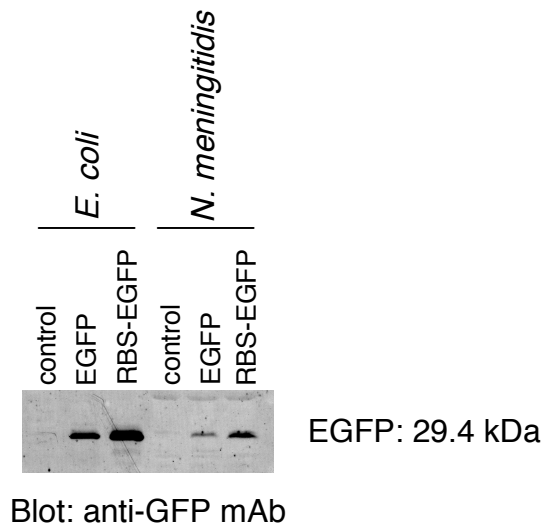

Fig. S3

Supplement: Figure S3 — Construction of a universal expression vector for N. meningitidis and expression of the egfp gene in N. meningitidis FAM20. A. The egfp ORF was PCR amplified from the pEGFP-C1 plasmid (Clontech) with the primers B1-egfp (AAA AAG CAG GCT ATG GTG AGC AAG GGC GAG GAG CTG TTC) and B2-egfp (AGA AAG CTG GGT TTA TCT AGA TCC GGT GGA TCC CGG G). To enhance the gene translation, an RBS-fused egfp ORF was also amplified with the primers B1-RBS-egfp (AAA AAG CAG GCT AGG AGG ATC CTA TGG TGA GCA AGG GCG AGG AGC TGT TG) and B2-egfp. RBS is underlined and the start codon of egfp gene is in bold. These two egfp containing fragment were cloned into pDONR 201, resulting plasmids pDONR egfp and pDONR RBS-egfp, respectively. The egfp ORF was assembled with the upstream region containing the porA promoter and the downstream fragment containing CmR after mixing plasmids pDONR P4-P1R 5-porA-pro, pDONR egfp (or pDONR RBS-egfp), pDONR P2R-P3 CmR-3, and pDEST R4-R3 as described in Fig. S2. The resulting expression plasmid was designated as pDEST-egfp or pDEST-(RBS)-egfp. B. egfp gene expression detected by western blot analysis. Plasmid pDEST-egfp or pDEST-RBS-egfp was transformed into E. coli DH10B and the N. meningitidis strain FAM20. Bacterial cell lysates were prepared and EGFP was detected using an anti-GFP monoclonal antibody (Roche). Parental bacterial strains were used as negative control (control). EGFP signal intensities from bacteria containing the additional RBS (RBS-EGFP) were higher than those lacking the additional RBS (EGFP) in both E. coli and N. meningitidis. (PDF) [file pone.0021749.s003.pdf]

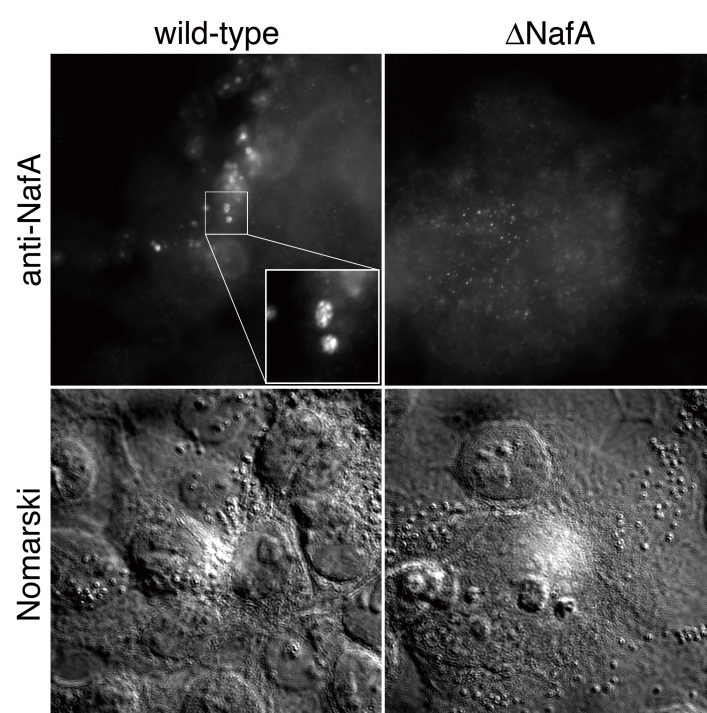

Fig. S4

Supplement: Figure S4 — Anti-NafA antibody prepared in present study is specific for NafA FaDu cells were infected with the wild-type strain (left column, identical photos in Fig. 2) or the ΔNafA strain for 6 h (right column). After washing away unbound bacteria, the cells were stained with anti-NafA peptide N antibody. Fluorescent (upper row) and Nomarski (lower row) images are shown. A 100 x objective lens was used. The inset shows higher magnification of the boxed area in the image. (PDF) [file pone.0021749.s004.pdf]
